# Design and Implementation of an ML and IoT Based Adaptive Traffic-Management System for Smart Cities

**DOI:** 10.3390/s22082908

**Published:** 2022-04-10

**Authors:** Umesh Kumar Lilhore, Agbotiname Lucky Imoize, Chun-Ta Li, Sarita Simaiya, Subhendu Kumar Pani, Nitin Goyal, Arun Kumar, Cheng-Chi Lee

**Affiliations:** 1 KIET Group of Institutions, NCR, Ghaziabad 201206, UP, India; umeshlilhore@gmail.com; 2 Department of Electrical and Electronics Engineering, Faculty of Engineering, University of Lagos, Akoka, Lagos 100213, Nigeria; aimoize@unilag.edu.ng; 3 Department of Electrical Engineering and Information Technology, Institute of Digital Communication, Ruhr University, 44801 Bochum, Germany; 4 Department of Information Management, Tainan University of Technology, 529 Zhongzheng Road, Tainan City 710302, Taiwan; 5 Institute of Engineering and Technology, Chitkara University, Rajpura 140401, Punjab, India; sarita.simaiya@chitkara.edu.in; 6 Krupajal Engineering College, BPUT, Kausalyapur 751002, Odisha, India; pani.subhendu@gmail.com; 7 Computer Science Engineering Department, Shri Vishwakarma Skill University, Palwal 121102, Haryana, India; er.nitin29@ieee.org; 8 Panipat Institute of Engineering and Technology, Panipat 132102, Haryana, India; ranaarun1.ece@piet.co.in; 9 Research and Development Center for Physical Education, Health, and Information Technology, Department of Library and Information Science, Fu Jen Catholic University, New Taipei City 24205, Taiwan; 10 Department of Computer Science and Information Engineering, Asia University, Taichung City 41354, Taiwan

**Keywords:** adaptive traffic management system, internet of things, machine learning, DBSCAN method, intelligent traffic management, smart road, intelligent transport system

## Abstract

The rapid growth in the number of vehicles has led to traffic congestion, pollution, and delays in logistic transportation in metropolitan areas. IoT has been an emerging innovation, moving the universe towards automated processes and intelligent management systems. This is a critical contribution to automation and smart civilizations. Effective and reliable congestion management and traffic control help save many precious resources. An IoT-based ITM system set of sensors is embedded in automatic vehicles and intelligent devices to recognize, obtain, and transmit data. Machine learning (ML) is another technique to improve the transport system. The existing transport-management solutions encounter several challenges resulting in traffic congestion, delay, and a high fatality rate. This research work presents the design and implementation of an Adaptive Traffic-management system (ATM) based on ML and IoT. The design of the proposed system is based on three essential entities: vehicle, infrastructure, and events. The design utilizes various scenarios to cover all the possible issues of the transport system. The proposed ATM system also utilizes the machine-learning-based DBSCAN clustering method to detect any accidental anomaly. The proposed ATM model constantly updates traffic signal schedules depending on traffic volume and estimated movements from nearby crossings. It significantly lowers traveling time by gradually moving automobiles across green signals and decreases traffic congestion by generating a better transition. The experiment outcomes reveal that the proposed ATM system significantly outperformed the conventional traffic-management strategy and will be a frontrunner for transportation planning in smart-city-based transport systems. The proposed ATM solution minimizes vehicle waiting times and congestion, reduces road accidents, and improves the overall journey experience.

## 1. Introduction

Nowadays, many things such as autonomous vehicles, collaborative transport systems, and intelligent roads are directly linked to IoT for ITM, enhancing data transmission and generating heterogeneous communication and low-bandwidth devices in large-capacity areas worldwide. India is a developing country, and its GDP was estimated to contract by 7.7% in the financial year 2020, compared to the growth rate of 4.2% in 2019 as per the report released by NSO [1]. This report proves that the economy of India has significantly improved and enhanced the living standards of civilians. The development of any country increases the number of personal and commercial vehicles. This has caused traffic congestion, a delay in logistic supply, a higher number of road accidents, and pollution. The demand for ITM systems has increased [2]. Mostly manual traffic controlling systems required higher manpower. These systems have desperately poor traffic policies and human resources strength, so authorities cannot manage traffic effectively in all cities using a manual system.

In order to solve traffic congestion, traffic signal systems are established in urban areas. However, the frequency division of traffic lights is equivalent and persistent for all the roads. Due to the dynamic nature of arrival traffic on all sides of the road, the signals are not equal, resulting in resource waste. As the volume of road infrastructures and automobiles grows, managing a traffic and transport network will be difficult. Generally, each road has a zebra crossing near the road signal, and each signal also has an assigned time to perform its function [3]. This complete process appears in a series. This traditional traffic-handling framework has a flaw in that it cannot detect the occurrence of automobiles across each route, and when a route is vacant, the traffic signal for that route squanders time. This traditional automobile management structure cannot handle traffic and control traffic jams. So, cities want a better alternative for the “Intelligent Transport Management System”.

An ITM system is a widely accepted system to overcome traffic management issues. ITM systems can reduce traffic congestion and enhance the quality of transportation for Logistics and passengers. The smartness and sustainability of the development of IoT-based ITM Systems depend on the solutions implemented to improve individuals’ standard of living. One crucial component of ITM strategies is smart-city governance, which creates planning methods for better policies. One of the core components of the smart-city management system is the public value of innovative services [4]. Over the last couple of decades, mobile appliances, sensors, and actuators have become more intelligent, facilitating communication between devices and the performance of complicated projects. Smart mobile devices, embedded systems, wireless sensors, and nearly all instruments are linked to a local network or internet leading into the IoT. Due to the increasing number of communication devices, the volumes of data generated by those devices also increase rapidly [5].

IoT includes linking physical things to the internet to create intelligent networks and mobile communication connectivity with innovative materials such as ITM. Communication among IoT-based automobiles is a new information-exchange paradigm contributing to ITM. IoT is a composition of data collection and analysis of sensor data and computing to effectively manage and support traffic networks [6]. On the other hand, automatic transportation containing a traffic signal utilizes a timer for each phase. The use of electronic sensors is another method of tracking automobiles. Although electronic traffic-control sensors have been used for traffic control, road traffic also happens. An intelligent transport system can resolve traffic congestion and other issues [7]. The primary aim of a smart city is to build a social structure that can accomplish the productive usage of urban services and infrastructure via AI and ML. This also concentrates on controlling the key characteristics, productivity, and enhancing the quality of resources for its community members [8]. Air quality and climate-change issues are essential research areas in smart cities. One research study [9] includes a viewpoint on the opportunities for highlighting urban air-quality management (UAQM) concerns by employing an intelligent urban model in ‘Smart urban computing.’ This research explains the responsibility of Intelligent urban computing in UAQM and offers a collaborative platform for smart urban computing environments for air pollutants processes.

Smart cities enable AI, ML, and IoT-based systems such as embedded linked devices, IoT sensors, smart traffic-control systems, smart streetlamps, and smart roads to gather and investigate statistics. These urban areas utilize this gathered information to optimize transportation systems, utility services, facilities, and creatures that interact with different platforms such as smart buildings, smart health care systems, smart automobiles, smart agricultural production, and Industry 4.0. This work presents an ATM based on ML and IoT design and implementation. The key contributions include:

1. The proposed ATM system utilizes the architecture and smart traffic signal to avoid congestion.
2. We introduce a completely deterministic adaptive technique for effective and close traffic monitoring and a congestion-control system at major regional intersections on any sequence of events.
3. One critical advantage of the proposed ATM structure is its ability to integrate with any adaptive method without requiring changes to the architectural style.

The complete research article is organized into various sections. Section 2 discusses existing work and covers a comparative analysis. In Section 3, materials and methods are covered; in Section 4, simulation experimental results and comparison of the results are covered. In the last section, five conclusions and future work are discussed.

## 2. Related Work

ITM boosts the ever-increasing vehicle motion and vehicular traffic in highway areas to avoid overcrowding. The generation of vast amounts of information produced by plenty of smart devices linked to the transportation system enables the formation of datasets that utilize deep learning algorithms to analyze data in depth.

### 2.1. Traffic Monitoring Based on Traffic Conditions

The authors of [10] explained an intelligent transmission-control system employing cloud perspective and ML methods. The graphics of its next vehicular intersection are captured and saved in the cloud database. The concentration and vehicle characteristics are identified using the cloud image API. The condition is also returned to the subsequent traffic signal. The prior traffic signal, which is now the current signal, will track the progress of the next traffic signal and then proceed with the activity predicated on the conditions. In order to increase the safety and efficiency of ITM, the authors of [11] demonstrated that these methods can help us to anticipate traffic performance, automated traffic-signal management, driveway identification, and recognition of nearby objects/vehicles. Various researchers are working on intelligent transport systems, but better traffic management is still challenging [12]. The authors of [13] demonstrated that significant traffic surveillance frameworks transform smart urban areas. Many studies have been conducted regarding intelligent traffic-control systems centered on the IoT approach.

The authors of [14] demonstrated that automatic traffic detecting is central to urban planning services and infrastructure. Intelligent connectivity sensor nodes estimate traffic flow, anticipate traffic jams, and adaptively control traffic movement. When performed correctly, this creates a level of consciousness that allows for more effective recourses and infrastructure usage. In another study [15], traffic flow, usage, and average density broadly utilized vehicular congestion assessments, most of which were acquired from pictures and videos recorded by computer vision applications. The authors of [16] proposed an IoT-enabled control system to gather, operate, and accumulate authentic traffic patterns for such a set of circumstances. Their primary goal was to deliver essential traffic information on road congestion and unexpected traffic collisions through the highway signaling system and increase the range of motion. The authors of [17] incorporated a framework to explore the effectiveness of the traffic model. The experimental findings demonstrate excellent vehicle detection and tracking precision and relatively low inaccuracy in highway occupancy prediction.

### 2.2. IoT Based Real-Time Traffic Management

Research has developed an IoT-based intelligent traffic strategy [18] to supervise significant congestion through centralized and decentralized domain controllers. The information-gathering component utilizes sensing devices, camera systems, and radiofrequency identification. Further, the application layer allows management of the traffic lights and notifications based on on-road vehicle frequency and offers a routine update through a software system. The authors of [19] described an inspection for reducing false projections based on the “Rankine-Hugoniot” circumstance and an origin–destination traffic facility. In order to authenticate the effectiveness of the suggested framework, a model was established. The testing results prove that the suggested method can successfully supervise precision and framework latency traffic congestion.

The authors of [20] used IoT-based linked vehicles to gather real-time data. The vehicle-to-vehicle connection supports individual vehicle surveillance, allowing precise collision-avoidance planning. The authors of [21] developed a perfected system for recognizing traffic patterns to configure on busy roads. The visual signal unit exhibits the ongoing traffic patterns and occurrences via notifications, indications, or color combinations. The authors of [22] suggested an expressive IoV routing protocol, recognizing complex relationships between automobiles, roadways, ecosystems, and pedestrian crossings. The authors of [23] developed an efficient solution to correct the park and ride solution based on the reservation-based optimal “park and ride” parking model inside the IoV ecosystem. The approach supports a layered approach to consciously considering vehicles by way of contact with the consideration of performance measures.

### 2.3. ML Methods in Real-Time Traffic Management

The authors of [24] presented a dynamic vehicular structure based on the IoT and ML concepts. Key responsibilities were played by the image sensor and two different control system panels. A scene detector mainly captured the specifics from the route with video coverage and transferred that to the following driver circuit. The authors of [25] compared multiple simulation models, a provisional logistic regression method, and a support vector machine method with predicting accidents. The method was evaluated relying on the information achieved from “Shanhai Middle Ring Highway,” China.

The authors of [26] suggested a method for predicting the volume of traffic, particular development stage vehicular region, and lane width. Researchers optimized the traffic-signal turnaround time and independent signalized intersections process time using the values produced by vehicles. The authors of [27] proposed a decentralized reinforcement learning-management system utilizing EA for a vehicular regulation system that efficiently improves the transport system’s efficiency. However, this was not incorporated at a certain period due to computing capacity restrictions. The paper also introduced a novel eco-friendly, flow-approximate solution that offered the traffic signal period for each straight path depending on the vehicular intensity. Then it used ML and the AI method to forecast the time duration in a small timeframe.

The authors of [28] used a methodology for intelligent water-quality tracking that focused on IoT. This research explored community water tracking criteria, hygiene for drinkable water, linked sensor technologies, critical evaluation, and the accession of the modern system via a proposed evidential measure, assessment, and discussion. Another research paper [29] discusses Air pollution monitoring using AI-based frameworks. This paper proposed an IoT-equipped climate-monitoring system for environment monitoring utilizing an artificially intelligent methodology to enhance biological life by overcoming the shortcomings of conventional monitoring systems and lowering overall costs. Research paper [30] discussed a two-step procedure for assessing the carbon productivity of urban integration hubs from well to vehicle. The carbon dioxide assessment of specific strategies is typically focused on a Tank-To-Wheel methodology that does not contribute to petroleum production and consumption, resulting in a preliminary evaluation of all its carbon impacts. The experimental results demonstrated that using the proposed monitoring and controlling system can save up to 190 tons of carbon dioxide.

### 2.4. VANET Based Real-Time Traffic Management

The author of [26], presented a VANET-based Smart ITM system. A VANET is a type of mobile ad hoc network in which intelligent automobiles on highways were also regarded as the connection point to communicate to transfer congested roads’ data. VANETs involve multiple network topologies, WLAN, an ad hoc network, and are adaptive. The authors of [31] proposed an advanced method to address this road traffic problem by using attributes of VANET. The mechanism is built and validated utilizing AODV procedures of mobile ad hoc networks to deal with vehicular road traffic in heterogeneous networks. The performance acted as an indicator through the number of transmissions distributed, the ratio of data packets, and a fraction of several vehicles rerouted and operational costs to handle the issues related to data congestion in-vehicle communication networks.

The authors of [32] utilized a Vehicle-to-Infrastructure interaction architectural design to adapt and incorporate two innovative solutions of an innovative “Intelligent Road Traffic Signaling” system and the “Predictive Road Traffic Signaling” system. The authors of [33] presented a distinctive VANET-enabled transportation and traffic signal management framework that significantly enhances vehicles’ movement, power efficiency, and the security of road users. In a research paper [34], a VANET-predicated technique was implemented utilizing a modular structure by integrating the interconnected connectivity characteristic. Research paper [35] recommends a distributed, interactive heavy-traffic detection and transmission system that utilizes VANET. A traffic app was installed on every one of the operators’ mobile phones to identify one’s position using the global positioning system.

### 2.5. Comparative Analysis of Existing Work

Table 1 presents a comparative analysis of various research works suggested by different researchers in IoT and ML-based intelligent transport systems.

## 3. Materials and Methods

### 3.1. IoT Architecture

The IoT defines the network of connected “things” that are often equipped with sensors, applications, and other advancements to integrate and transfer information between devices and platforms over the Web. The IoT has two main components. The first is an “object or thing” which users intend to make intelligent through interconnection, and another is the embedded platform that enables this communication. The latter part may seem easy, but consists of a complicated structure composed of various sensors, actuators, methods, and data-access layers. Each interconnection is accountable for creating configurable, intelligent, and successful connections with human beings [51].

**Figure 1:**
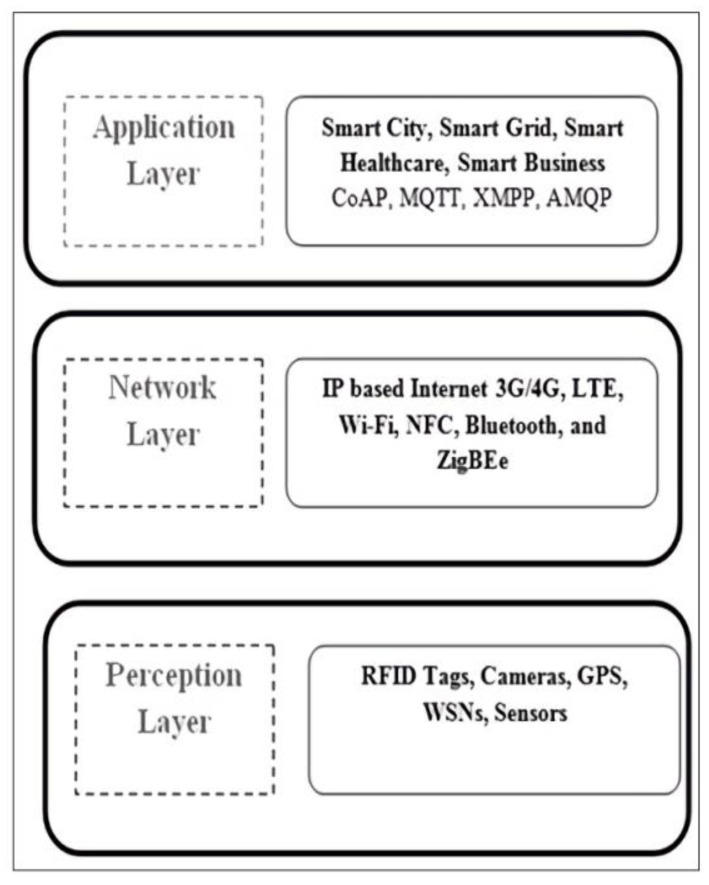
The layered architecture of IoT.

Figure 1 shows the three-layered architecture of IoT. This model’s first layer (bottom to top) represents the perception layer, including IoT components, i.e., sensors, GPS., RFID tags, and cameras. The application layer includes various protocols CoAP (Constrained Application Protocol), MQTT (Message Queuing Telemetry Transport), XMPP (Extensible Messaging Presence Protocol), and AMQP (Advanced Message Queuing Protocol), which provides application in the field of Smart City, Smart Grid, Smart Healthcare, and Smart Business. The second layer is the network layer, which mainly represents communication technology and media, i.e., internet type (3G/4G), medium, and communication type. The top layer is the application layer representing the final application or end-user viewing the IoT communication.

### 3.2. IoT in ITM

**Figure 2:**
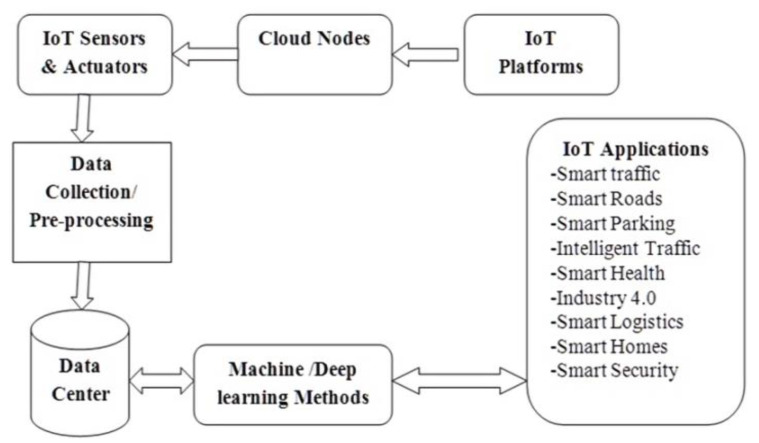
IoT application in ITM.

The rise of the IoT technique and the availability of cloud resources are supporting us in establishing processes that can integrate the transport systems and enhance the use of current facilities. An ITM system utilizes the essential features of well-known technologies, including IoT, cloud computing, ML, AI, and big data. In IoT, the objects need to interact with communicating devices using M2M (Machine to Machine). The introduction of the IoT and its relevance in transport systems creates the perfect platform for acknowledging traffic-related difficulties, consequently leading to the formation of an ITM [52]. Figure 2 shows the use of IoT in ITM. Each component is interconnected with other component blocks. The IoT components include sensors, actuators, internet platforms, cloud nodes, data centers, and ML methods.

In Figure 2, various blocks show how the IoT-based system helps in ITM. The first block shows the IoT platform, which includes components, i.e., sensors and cameras, which mainly collect the data from the traffic environment and transfer these data to the cloud. The cloud node mainly stores the data. These blocks represent the complete data-collection process of an IoT-based system. Additionally, IoT sensors and actuators collect data from moving objects such as vehicles. The completed data are transferred in preprocessing blocks in the next phase, which removes the various anomalies from the dataset. These data are mainly stored in data centers. Later the stored data help in various fields of ITM. Various Machine and deep-learning-based models are utilized to retrieve these data centers’ information.

### 3.3. Proposed ATM System Design and Implementation

**Figure 3:**
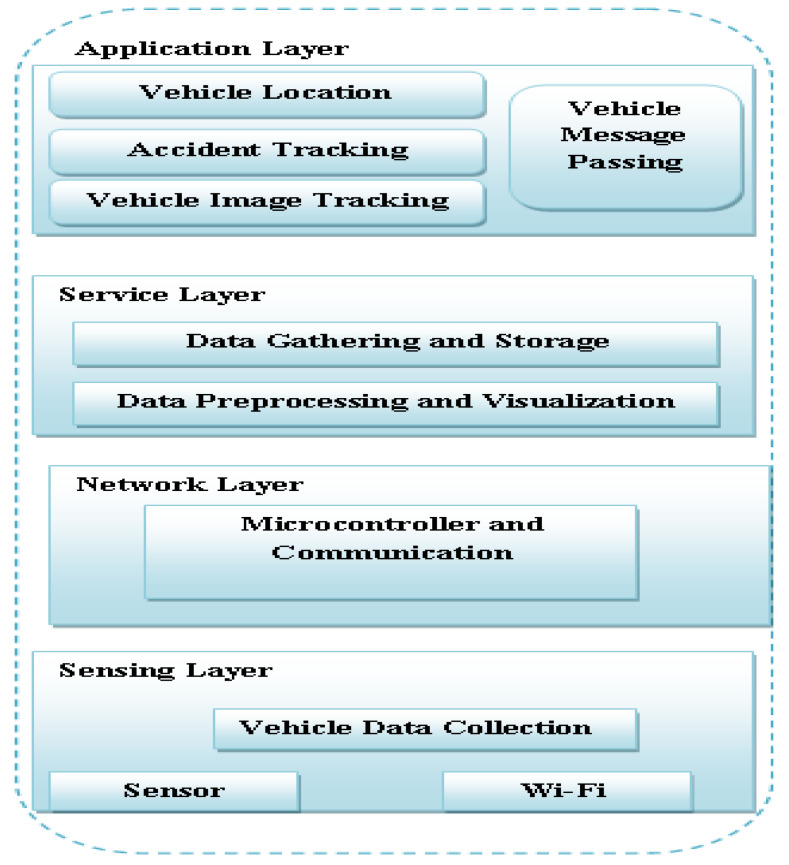
Layered architecture of the proposed ATM model.

The proposed ATM system involves the promising approach of the intelligent transport system to address the actual significant problem in management traffic. The proposed ATM model utilizes the following modules to develop an intelligent transport management system. Figure 3 shows the layered architecture of the proposed ATM model. The first layer is the application layer, representing the vehicle’s location, accidental tracking, message passing, and image-tracking details. Layer 2 is the service layer, representing the data gathering and storage process and showing the data preprocessing. Layer 3 is the network layer, representing the communication, and Layer 4 represents the sensing layer.

#### 3.3.1. Vehicle Location Tracking

**Figure 4:**
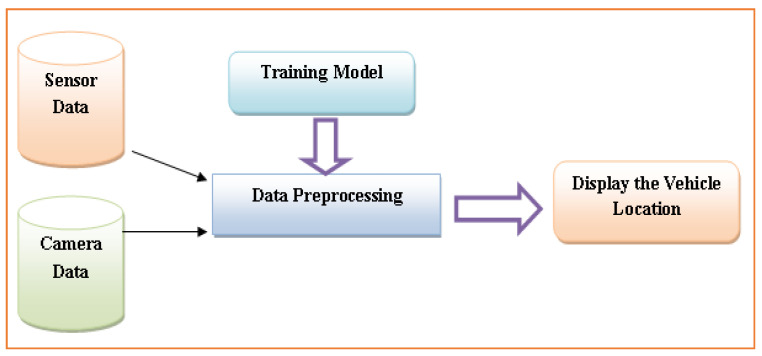
Vehicle Location Tracking in ITM.

The proposed ATM system helps choose those routes that provide higher precision. The model is validated for its performance with the benchmark’s lower bound precision value. Still, suppose the proposed model generates the desired precision for the lower bound. In that case, this strongly implies there are good effective routes, and all the other lesser communication routes are removed. However, if the lower bound is more than the predicted precision rate, there are not enough routes. The critical paths for effective vehicle localization are further added to the set. Figure 4 displays the functioning model of the proposed vehicle location tracking system module. In the first phase, data are collected using the sensor and camera devices. The capture of data by sensor and camera and the preprocessing of those data are vital components in ITM. The missing value estimation methods are used in the preprocessing data phase [53]. The processing method processes these collected data and later applies the training method to train the dataset. The vehicle’s exact location and traffic details are collected.

The feature clustering is achieved by creating a graph to prevent it. The nodes represent feature paths, the edges represent path clustering interactions, and the network nodes (feature groups) represent vehicle observations [54]. For each feature and attribute at a time interval (*T_i_*):

- Step 1—Features identified at a time interval (*T_i_*) for the frame (*F_i_*) are picked and monitored for a threshold number of frames, if the expressed cumulative personal motion is sufficiently massive. Almost every newly formed feature that is extracted is linked to the presently recorded characteristics inside an Euclidean distance minimum.
- Step 2—The distance (*Dis_i_*_,*j*_) between all presently monitored sets of linked functionalities (*Lf_i_*_,*j*_) is approximated, and the upper and lower limits intervals are revised. The *D_seg_* represents the value of the feature segmentation threshold. The linked vehicles’ characteristics can be defined as mentioned in (1).
  [*Max T_i_d_ij_* (*T_i_*) − *Min T_i_d_ij_*(*T_i_*)] (*D_seg_*) (1)
- Step 3—The graph’s linked features are discovered. Each related component, i.e., pair of attribute paths, represents a vehicle observation. Suppose all of the functionalities that comprise a factor are no longer recorded. The attributes are eliminated from the graph, and the vehicle hypothesis’ attributes (speed vector, centroid position, and vehicle size) are calculated.

#### 3.3.2. Accident Detection Module

An ITM system can reduce the possibility of accidents and the number of accidental deaths by applying intelligent traffic control. The proposed ATM system helps the road accident be detected by the fall of the automobile using an intelligent accelerometer sensor. An accelerometer sensor mainly measures the speed forces in which it is equipped. These forces may also be fixed, such as the constant gravitational pull or a scenario with many smartphones, which can detect mobility or motions. Acceleration is the standard measure of the change in speed or velocity differentiated by time period [54]. This model is mainly performed using the ML method. The scheme is trained using preprocessed vehicular accident samples, and the training is completed using pressure and distance. The pressure is identified by utilizing data from sensing devices, where distance is evaluated with a U.C. sensor. A motion sensor determines speed, and volume is calculated with a load-sensing element. The formula for calculating force using kinetic energy and work is mentioned in Equation (2).

*W_force_* = (*K ∗ E_energy_*) (2)

#### 3.3.3. Vehicle Image Processing Module in ITM

This model first identifies the motor vehicles via images and electronic sensors integrated into the road surface. A webcam will also be positioned along with the traffic signal and sensors. This will capture patterns of image data. Object detection is the best alternative to regulate the state change of the traffic signal. It can reduce road-traffic congestion and minimize wasted time with a green light over an empty highway. Furthermore, it is more accurate in predicting vehicle presence because it utilizes actual traffic-image data. It analyzes the usefulness and processes much better than all those models that depend on identifying the vehicular surface material [55]. Algorithm 1 represents the working of Image processing in the intelligent transport system.
**Algorithm 1:** Image processing in the intelligent transport systemStep-1. **Image data collection:** using a camera and sensor installed over the road.Step-2 **Preprocessing phase:** To process the images as follows-      2.1 Images are converted into a standard size (i.e., 450 × 450 pixels)      2.2 Convert all the captured RGB images into grayscale images.Step-3. **Edge detection phase:** Canny edge detection methodStep-4. **Pixel match technique:** The output of step 3 is compared by using pixel to pixel (P.P.M.) matching techniquesStep-5. **Timing allocation:** It depends on the result of step 4; the percentage of image matching criteria is as follows:      5.1 If the image matched = 40%, then on a green light for 90 s      5.2 If the 40% image matched = 70% then on green light for 60 s      5.3 If the 70% image matched = 90% then on green light for 30 s      5.4 If the 90% image matched = 100% then on Red light for 90 s      5.5 Repeat steps 3–5

#### 3.3.4. Vehicle Communication with VANET

The sensors monitor the vehicle’s position in the current traffic situation by registering the motor vehicle and its communications equipment. Through the use of IoT sensor systems, data are transmitted and distributed among vehicles in such a way that helps prevent traffic and ensure safe travel [56]. The platform design is created to notify ahead of the vehicular situation and unsafe driving scenarios and handle the unpredicted injuries and deaths and scenarios with statistics to be acknowledged ahead to vehicles on the road for secure traveling. It is also necessary to communicate the messages to the drivers by extracting the incredibly massive amount of past reliable statistics based on current traffic conditions [57]. The Roadside Unit (RSU) plays a vital role in communication in VANET. Algorithm 2 represents the working Vehicle communication process in proposed ATM.
**Algorithm 2:** Vehicle communication process in proposed ATMStep-1 installed the RSU unit set the roadside at a specific distanceStep-2 Vehicle connection setup with RSU       2.1 Neighboring vehicle receives a setup connection request from RSU       2.2 Vehicle sends the required data, i.e., location, velocity, start time to RSUStep-3 Data storage: RSU stores all the received data in a data-basedStep-4 RSU Interval: if RSU received more than one request from multiple vehicles, then apply the wait and synchronization method for data storage per the time interval.Step-5 call (Image processing in ITM) method is described in the previous section.Step-6 Vehicle synchronization: if Synchronization values are high (because of higher speed vehicle), send the alert data (priority)Step-7 Eliminate vehicle: remove the low-velocity vehicle and set the lower priorityStep-8 RSU communication: RSUs communicate with each other and share alert messages to handle congestion

#### 3.3.5. Machine Learning in ITM

**Figure 5:**
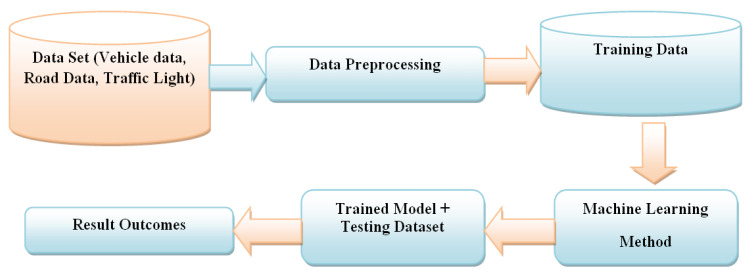
Vehicle location tracking process in ITM (using DBSCAN Clustering method).

Intelligent transportation is a scorching research area because it discovers numerous real-world issues, with massive infrastructure in the advent of the smart metropolitan areas [58,59,60]. Furthermore, the conflicts it handles with favorable treatment utilize IoT and ML technological advances (Figure 5). The dataset includes the complete details of the traffic environment. It includes vehicle data, road data, and traffic details.

Algorithm 3: The proposed ATM model utilizes the machine-learning-based DBSCAN clustering method. This method deceived the MATLAB traffic simulator results and clusters into detecting any accidental anomalies.
**Algorithm 3:** DBSCAN (D_a_, minimum_points, epsilon)// Detection of a vehicle collision on the roadInput: dataset accidents D_a_; clusters C_k_; and cluster mean M_c_Output: accidental cluster groups recognize C_ki_
      Step-1 initialize the cluster C_k_ = 0       Step-2 Mark all the unvisited entries U_.D._ as visited VD in the dataset       Step-3 Calculate the s_p,       Where s_p is sphere_points, m_p is min_points, and r_Q is region_Quer.       s_p = r_Q(V_D_, epsilon)       Step-4 if size of (s_p) m_p) not consider the value of V_.D._
      Else       Step-5 Calculate the next cluster by       C_k_ = C_new_, where C_new_ is the next cluster value       Step-6 Call the expand clustering function E_C ( )       6.1 E_C(V_D_, s_p, C_k_, epsilon, m_p);       6.2 E_C(V_D_, s_p, C_newi_, epsilon, m_p);       Step-7 Add all the new visited V_.D._ to cluster set C_k_
      Step-8 Verify for all the points V_.D._ in s_p       Step-9 For instance, if V_.D._ is marked as unvisited       Step-10 Update the V_.D._ and set it as status visited       Step-11 Calculate s_p=r_Q (V_D_, epsilon)       Step-12 Verify the size by if size of(s_p) = m_p       Step-13 s_p = New s_p U existing s_p       Step-14 for any of the instances if V_.D._ is not in any of the cluster set       Step-15 update the V_.D._ status and add V_.D._ to the C_k_ cluster                   15.1 Calculate the region are and execute the r_Q()                   15.2 R_Q(V_.D._, epsilon);                   15.3 Return all the new points inside the n-dimensional V_.D._ towards the radius epsilon.

### 3.4. Mathematical Model of the Proposed ATM System

The proposed ATM model is based on “Platoon-based traffic flow”. A Platoon is usually interpreted as a cluster of automobiles moving next to each other, willingly or unwillingly [61].

**Figure 6:**
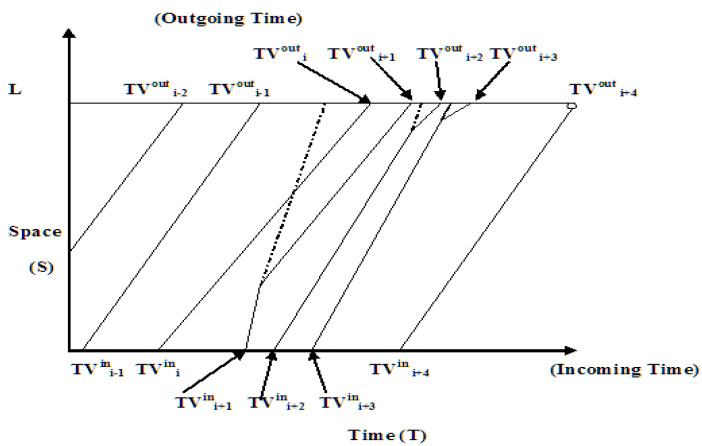
Traffic flow space vs. time (incoming and outgoing vehicles).

In the proposed ATM’s mathematical model, every vehicle is anticipated to get an impartial requested power V, and the traffic variation, FT (V), is provided. If it closes the gap to the traffic ahead, automobiles move constantly. After catching up, the object instantly reduces its motion to that of the moving vehicle and continues to follow it, making constant progress. Figure 6 shows the traffic flow based on available space v_s_. time. L represents the length of the road lane. TV^In^ represents the entering time, TV_out_ represents the out time of vehicle, V_i_, and (TVh_i_) represents the headway time of vehicle, V_i_ mentioned in Equation (3).

(3) $$T^{\mathrm{out}}=\{\begin{array}{c} {\mathrm{TV}}^{{\mathrm{out}}_{i}}\;\left(for\;i=0\right)\\ Max\;({\mathrm{TV}}^{{\mathrm{out}}_{i}},\;{\mathrm{TV}}^{{\mathrm{out}}_{i-1}}+{\mathrm{TVh}}_{i}\;\left(for\;i=1,2,\ldots ,n\right) \end{array}$$

The time-out TV^out^, can be calculated by Equation (4). When i = 0, there are no automobiles next to the boundary configuration.

TV^out^ = {TV^in^_i_ +(L/V_i_)} (4)

Let PV (EV_s_) be the deterministic probability of a platoon of N automobiles trying to form the frequency of event EV_s_. Here, E is the event, and PV (N|EV_s_) is the deterministic probability of a platoon of N automobiles providing the occurrence of event EVs. Finally, at the end of the evaluation portion, the probability PV^PrimaryV^ (N) that such a specific new vehicle will become the prime vehicle of a platoon of n automobiles mentioned in Equation (5).

PV^PrimaryV^(N) = lim_0-∞_ PV (EVs) ∗ PV (N|EVs) ∗ F_V_(PV)dv (5)

The results of PV (EVs) and PV (N|EVs) can be determined by the speed distribution mapping (Figure 6).

#### Mathematical Model Formulation for Proposed ATM

Let the vehicle congestion deterministic model CDM in a platoon of vehicles (V_i_) on the road (R_i_) be (I = 1…n). The Markov speed (M_speed_) property of any vehicle in a platoon. State space can be defined as {S_T0_…S_Tn_). A matrix (M) for state space can be defined as mentioned in Equation (6).

$$\begin{array}{cccc} M=\\ \text{M }S_{\text{T0}}{\text{ S}}_{\text{T0}} & \text{M }S_{\text{T0}}{\text{ S}}_{\text{T1}} & \ldots & \text{M }S_{\text{T0}}{\text{ S}}_{\text{Tn}}\\ \text{M }S_{\text{T1}}{\text{ S}}_{\text{T0}} & \text{M }S_{\text{T1}}{\text{ S}}_{\text{T1}} & \ldots & \text{M }S_{\text{T1}}{\text{ S}}_{\text{Tn}}\\ \ldots & \ldots & \ldots & \ldots \\ \text{M }S_{\text{Tn}}{\text{ S}}_{\text{T0}} & \text{M }S_{\text{Tn}}{\text{ S}}_{\text{T1}} & \ldots & \text{M }S_{\text{Tn}}{\text{ S}}_{\text{Tn}} \end{array}$$ (6)

Here, a transition probability from the state P to Q can be defined as M_PQ_. The top-speed vehicle on the road can be defined as TV_top_. The state vector (B^(i)^ TV_top_) for any ith vehicle mentioned in Equations (7)–(9).

(B^(i)^ TV_top_) = (B^(0)^ TV_top_)*A^(i)^ (7)

(B^(0)^ TV_top_)* = (Ώ_0_, Ώ_1_, … Ώ_n_)^T^ (8)

(9) $$\Omega_{n}=\{\begin{array}{c} 1\;\left(for\;{\mathrm{TV}}_{\mathrm{top}}\in \;S_{\mathrm{Ti}}\right)\\ 0\;\left(for\;rest\;all\right) \end{array}$$

## 4. Discussion

The proposed ATM system was implemented on MATLAB simulator. The proposed system utilizes three primary entities (vehicles, infrastructure, and events) described in Table 2.

**Figure 7:**
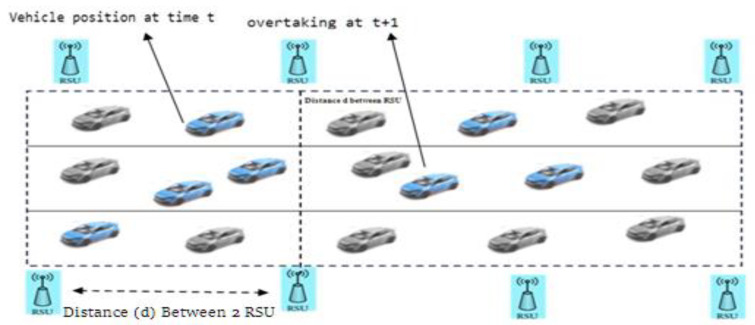
System Design of proposed ITM.

In order to validate the efficiency and accuracy of the proposed ATM system, various traffic scenarios were created for Linked automated vehicles (LAVs) as follows. (a) Only with linked LAVs (b) Where Only with Non-LAVs, (c) in which for LAVs and Non-LAVs, both types of vehicles are moving. Figure 7 shows the system design of the proposed ATM model for road-traffic conditions on the road with both the moving vehicles in a forward position.

(a) **Only with LAVs**—This is the first scenario considering only LAVs. In this scenario, the intelligent traffic-management systems mainly divide the traffic into two segments. The first is the control segment (CS), and another is the merging segmentation (MS). The CS has a control entity named control unit (CU), which helps it to communicate with LAVs [62].
(b) **Where Only with Non-LAVs**—Assessments are necessary to verify the effectiveness of proposed ATM methods. As a result, a traffic virtual environment system must be easily adaptable to various traffic situations, allowing users to compare diverse perspectives. A baseline sequence of events is developed and evaluated on the vehicular modeling in which just the fixed-cycle traffic illumination monitors the Non-LAVs [63].
(c) **Where LAVs and Non-Linked both types of vehicles are moving**—The mixed-traffic case, in which both LAVs and Non-LAVs move on the roadways, should be viewed as a significant challenge for the massive implementation of automated vehicles. System model control techniques are tested on the proposed approach for this situation. Figure 5 shows the results for LAVs and Non-Linked automated vehicles [64].

IoT devices will collect the live vehicle-traffic data and road conditions and store these data in the data center, implementing the proposed ATM system. In the next phase, big data techniques will apply to process the live data. The final phase will be based on ML methods to train the data. We assume that the motor vehicles are fully equipped with a wireless system module that interacts with the RSU in the proposed ATM system. (Figure 7) positioned mainly on the highway to transfer heavy traffic data with other moving automobiles.

In contrast, automobiles are assumed to be adequately equipped, mainly with the entire event query recorder (EQR) used to measure an automobile’s fast movement speed and traffic details. In the proposed system, the roadside units are positioned on the road at a range of approximately 1.0 km apart. All such roadside units are ready to supply fair coverage in their region and are located by the RSUs nearby. Those on the right side of the road are also used to build architecture. Almost every RSU includes a GPS unit to acquire the precise location of automobiles, a transceiver for creating communication among passing traffic, and a computing device that delivers live traffic data gathered from automobiles, such as lane-altering distance and over speed [59,60].

**Figure 8:**
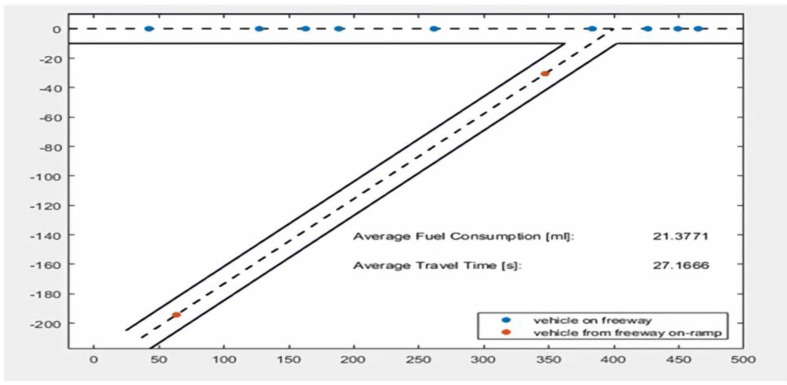
Vehicles moving on the freeway where fuel less fuel consumption, and average less travel time is generated by the travel report.

**In scenario: 1** (Figure 8), vehicles move in one direction over the road and move from the freeway. In this scenario, one vehicle consumes less fuel and travel time.

**Figure 9:**
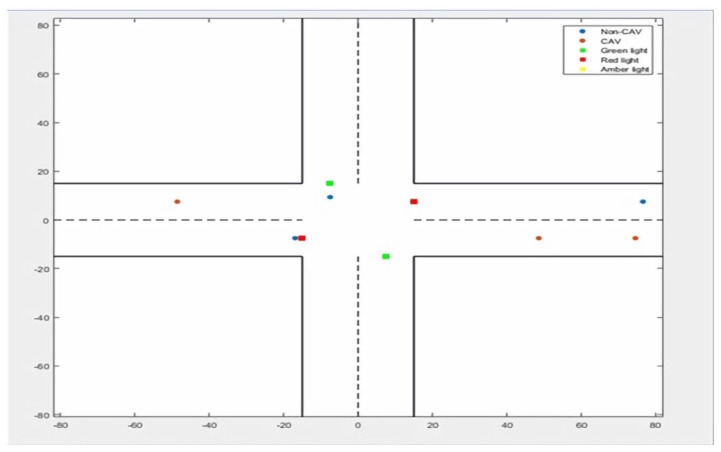
Vehicle moving on the freeway (multilane), connected/linked and automated vehicle, and signals in 3 categories (red, green, and yellow).

**Figure 10:**
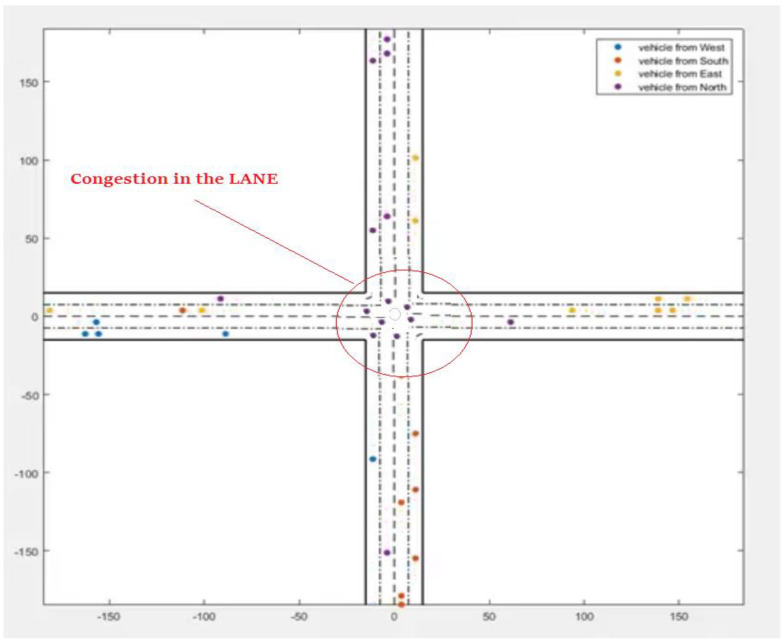
Vehicle moving on forward and backward over multiple lanes (traffic congestion).

**Figure 11:**
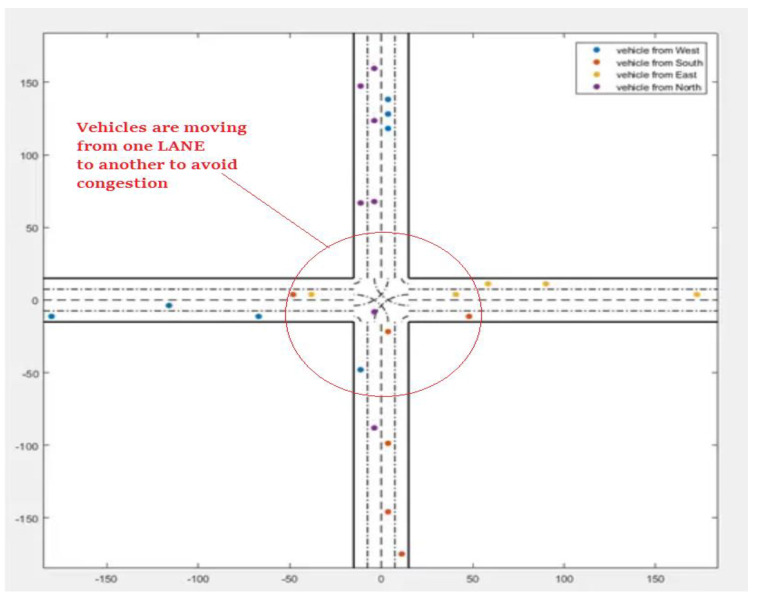
Vehicle movement based on signals received from RSUs.

In the second scenario, vehicles move backwards and forward over a multilane path (Figure 9). In scenario 3, vehicles move forward and backwards over multiple lanes; this is caused by “Traffic Congestion,” as described in Figure 10. The traffic congestion is resolved by the proposed intelligent traffic-management system described in Figure 11.

### Experimental Results and Discussions

The simulation results demonstrate that the proposed ATM system outperforms the conventional traffic-management system in packet delivery ratio, throughput, and time delay [60]. This serves best in case of a change in traffic conditions (as per scenarios 1 to 3) and latency. The graphs below accurately depict the numerous criteria and significance regarding particular system strategies for correspondence with data packets. The experimental results were performed on MATLAB simulation with 100 vehicles, a 5000 m road, and different moving directions (i.e., Channel 1: East to West; Channel 2: North to West; Channel 3: North to East; and Channel 4: East to North). In all the simulations, traffic is moving in inflow and outflow directions. The model type is the Random model. The probability for inflow traffic is 0.58%, and for outflow it is 0.49%. The simulation results were calculated for three different scenarios.

**Figure 12:**
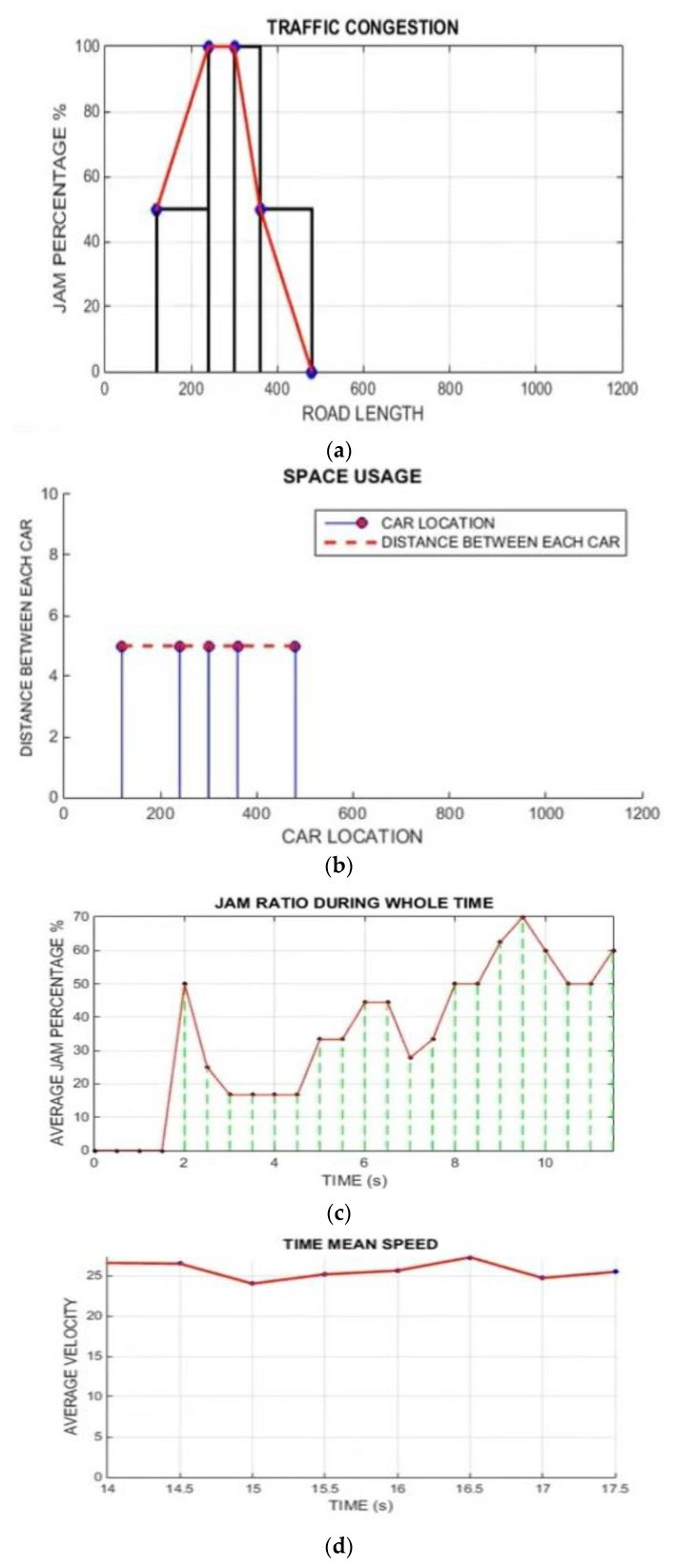

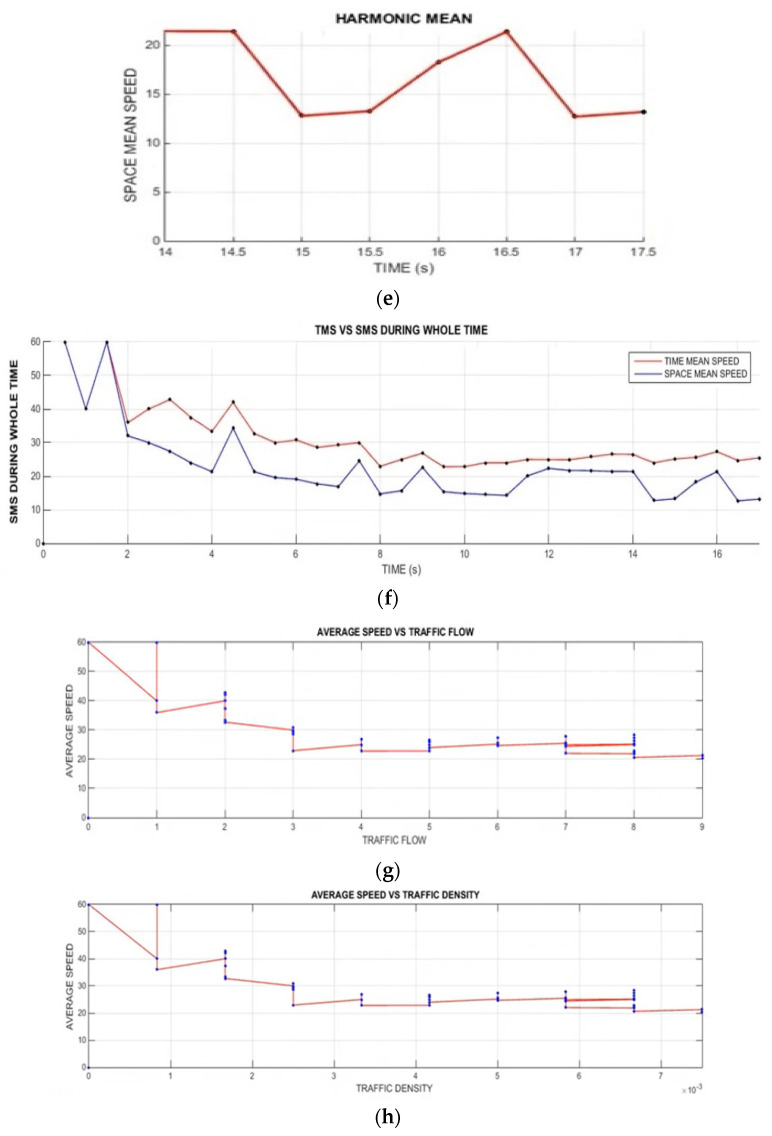
Simulation results for scenario 1 only with LAVs (From Figure 12a–h). (**a**) Traffic congestion; (**b**) space utilization; (**c**) traffic jam ratio; (**d**) time mean speed; (**e**) harmonic mean; (**f**) time means speed vs. space means speed; (**g**) scenario 1 results for average speed vs. traffic flow. (**h**) Scenario 1 results for average speed vs. traffic density.

Figure 12 shows the simulation result for scenario 1 only with LAVs. Figure 12a shows the traffic congestion ratio, depending on road length and traffic-jam percentage. Figure 12b shows the space-utilization graph, which depends on the car location and the distance between each car. Figure 12c shows the traffic-jam ratio results, which depend on the jam ratio during the whole time and the average jam percentage. Figure 12d time mean speed; Figure 12e harmonic mean; Figure 12f time mean speed vs. space means speed results; Figure 12g average speed vs. traffic flow and Figure 12h average speed vs. traffic density.

**Figure 13:**
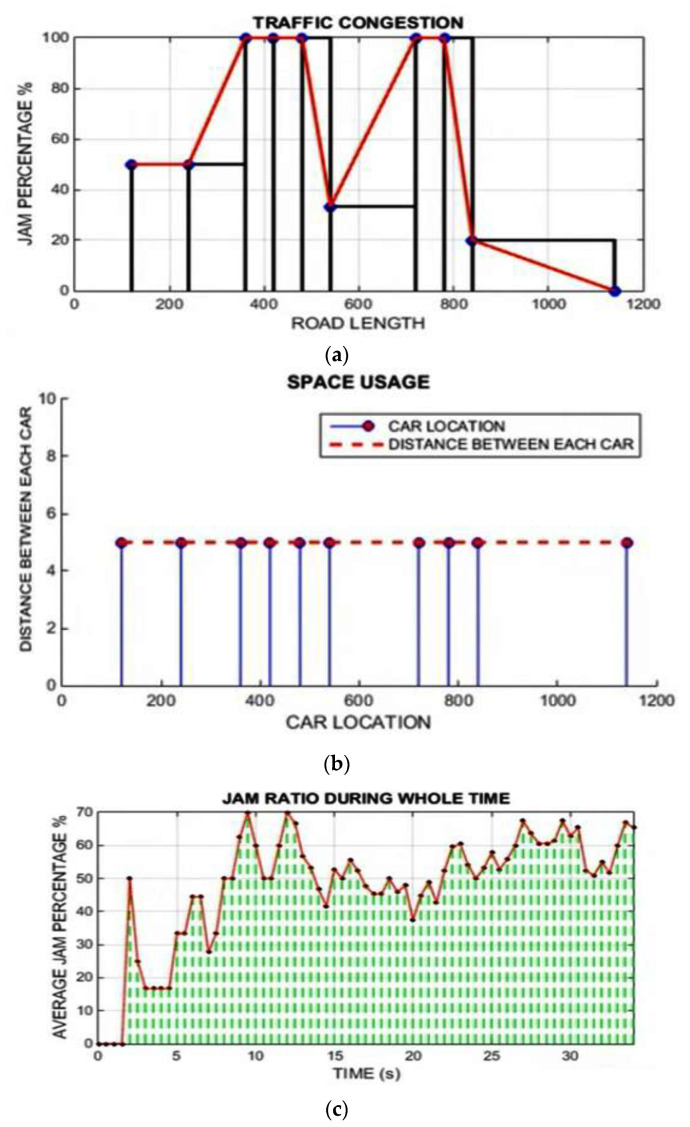

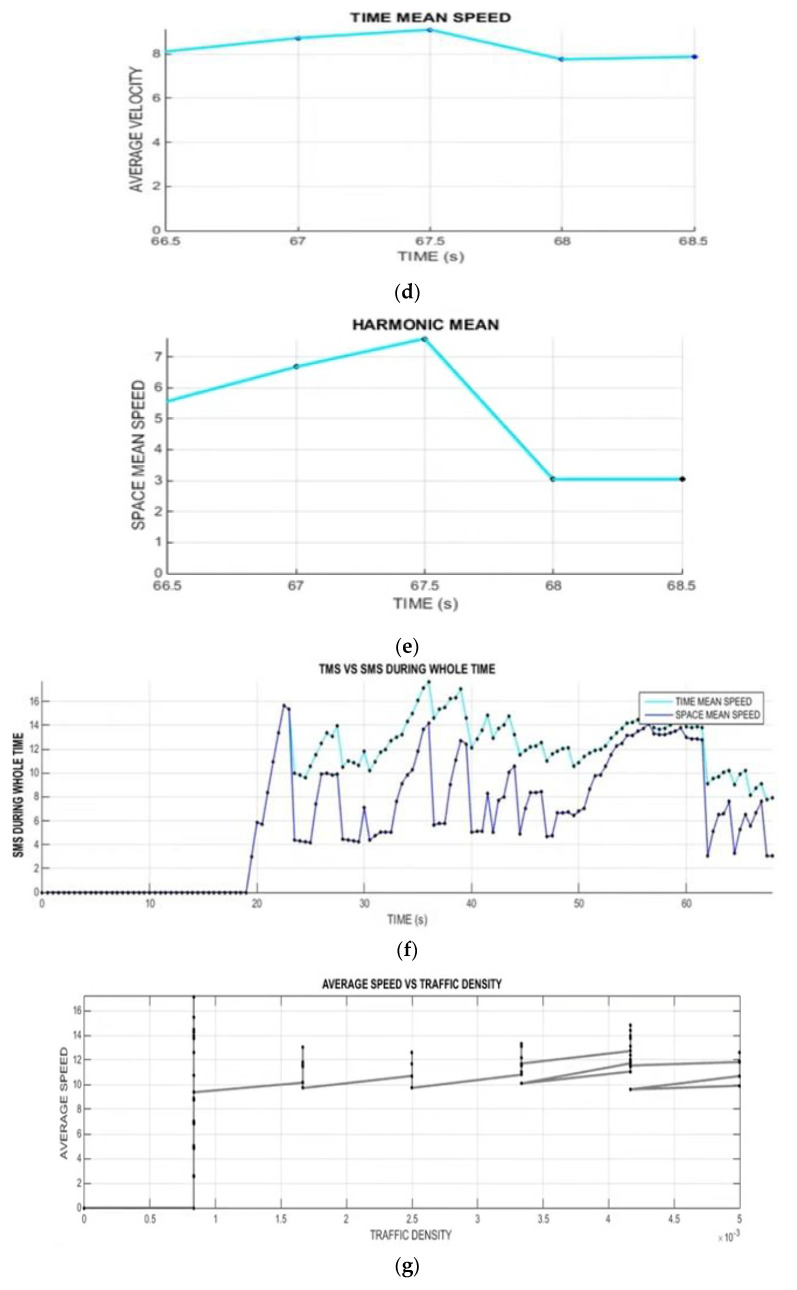

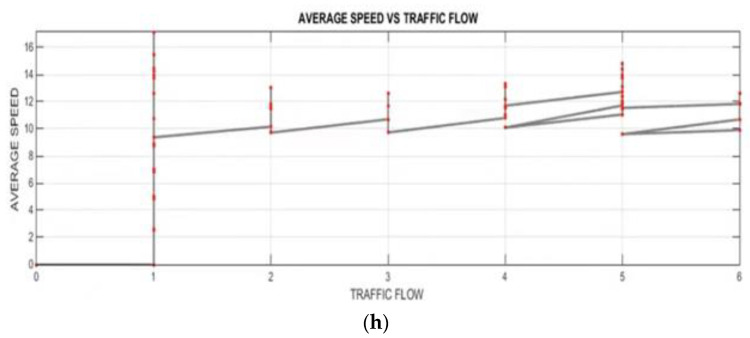
Simulations results for scenario 2, no-LAVs vehicles (from Figure 13a–h). (**a**) Traffic congestion; (**b**) space utilization; (**c**) traffic jam ratio; (**d**) time mean speed; (**e**) harmonic mean; (**f**) simulation results for TMS vs. SMS during the whole time; (**g**) simulation results for scenario 2 average speed vs. traffic density; (**h**) simulation results for scenario 2 average speed vs. traffic flow.

Figure 13 shows the simulation result for the second scenario, which is related only to Non-LAVs. Figure 13a shows the traffic congestion ratio, which depends on the road length and traffic-jam percentage; Figure 13b shows the space utilization graph, which depends on the car location and distance between each car; c shows the traffic-jam ratio results, which depend on the jam ratio during the whole time and average jam percentage %. Figure 13d Time mean speed; Figure 13e harmonic mean; Figure 13f time mean speed vs. space mean speed results; Figure 13g average speed vs. traffic flow and Figure 13h average speed vs. traffic density.

**Figure 14:**
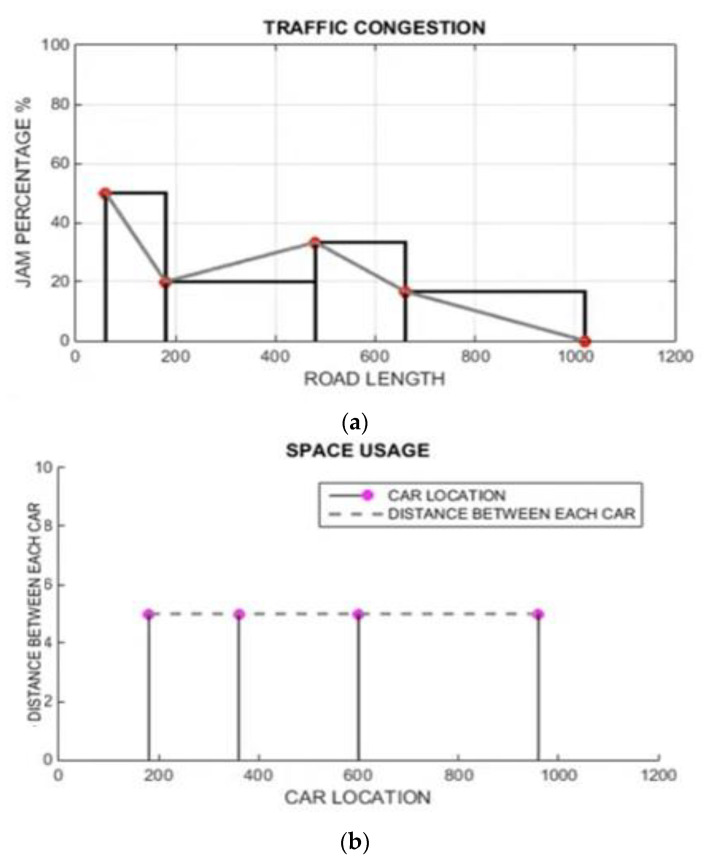

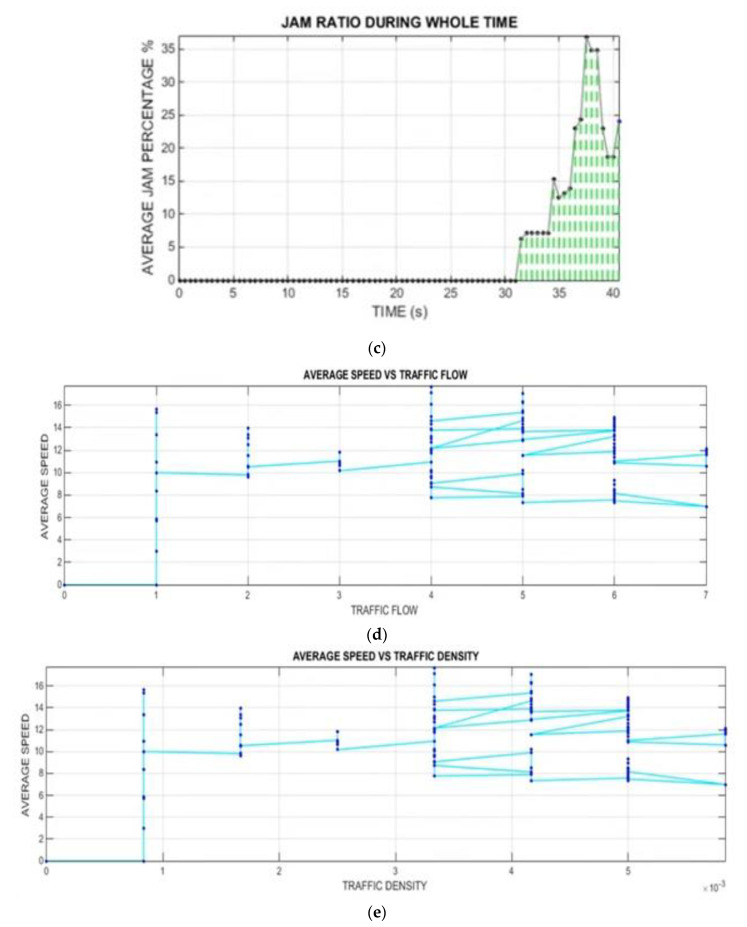
Scenario 3: Hybrid simulation (LAVs and no-LAVs) vehicles results. (**a**) Traffic congestion; (**b**) space utilization; (**c**) traffic jam ratio; (**d**) average speed vs. traffic flow results; (**e**) average speed vs. traffic density results.

Figure 14 shows the simulation result for scenario 3, Hybrid with NAVs and Non- LAVs. Figure 14a shows the traffic congestion ratio, which depends on the percentage of road length and traffic jams. Figure 14b shows the space utilization graph, which depends on car location and the distance between each car. Figure 14c shows the traffic-jam ratio results, which depend on the jam ratio during the whole time and the average jam percentage %. Figure 14d Average speeds vs. traffic flow and Figure 14e average speeds vs. Traffic density.

MATLAB simulator is a collision-free traffic simulator. In order to detect the accident in the next phase, a ML-based DBSCAN method will apply. Table 3 represents the Clustering Outcomes of DBSCAN and ML methods for accident detection. A vehicle is pressured to come to a halt in a predetermined location. Halts can also be viewed as significant incidents in a section of the road. Vehicles on their own or divers driving the vehicle and passengers traveling in the vehicle can also make the transportation system difficult. Recognizing such instances and activating coming automobiles will avoid potential collisions. At each 100 s timeframe of the simulation, each vehicle is required to halt at a road view. In order to achieve a remarkably rapid stop, the deceleration point is fixed to 60 m/s^2^. In this simulation, we are using three types of vehicles.

The proposed ATM system helps with effective vehicular tracking. It also aids inconvenient traffic congestion so the motor vehicles are redirected in a congestion scenario in a particular place. The traffic conditions, density, and traffic-flow criteria predict the traffic situation, and automobiles are redirected to their destination without further accidents. The vehicle density is lower with the rise in range for the proposed ATM system as the current scheme tries to resolve road traffic without too many difficult situations. The automobiles are also redirected to the alternative route to prevent congestion. The proposed ATM system significantly outperformed the other existing structure. Apart from previous designs, the proposed ATM accepts different countries’ traffic situations and traffic movement patterns and communicates in actual environments. It supports day-to-day operations with downstream monitoring and a consumer environment. In terms of achieving highway interchanges green–green coordination throughout the distribution zone, traffic information from crossings is aggregated in a centralized transport network. Advanced traffic control algorithms generate optimum red–green cycles of traffic lights. In live time, the ATM constantly reacts to changing traffic situations. ATM analyzes real-time traffic information from automobile detection using a machine learning approach to calculate the signal durations that are best for current traffic situations.

## 5. Conclusions

This research provides an ITM system for tracking LAVs and Non-LAVs vehicles related to potential highway-vulnerability factors. The proposed ATM model enables in-location services of automobiles, parking management, and implementing traffic-management techniques for development of an intelligent transport system. The scheme helps to monitor automobile movement, thereby examining the traffic in a specific region. 

Automatic accident detection has become a popular topic in vehicular traffic-management systems. Surveillance of an accident can help us to avoid possible similar incidents in the future, and it will facilitate security agencies in reopening the road segment to a number of vehicles. We successfully demonstrated that vehicular activity could be evaluated, utilizing vehicular locations and average speeds. Additionally, abnormal events on the highway can be considered a future challenge for drivers who have already been nearest to the accident region. It was found that the proposed ATM system had a superior performance to the existing conventional systems.

Future work will integrate energy-efficient systems and security into the proposed ATM system. The proposed system will be implemented in a real-time environment in the place of the simulator with real-time traffic flow.

## Figures and Tables

**Table 1:** Review of various research in IoT and ML-based intelligent transport systems.

Ref. No.	Key Technique	Methods/Algorithm	Traffic Congestion	Smart Parking/Road	Merits
[36]	Traffic congestion detection	Machine learning, IoT	Yes	No	Automatic vehicle detection method and automatic route-transfer method
[37]	Collision avoidance	IoT, Big data	Yes	Yes	Design collision-free protocol for transportation
[38]	Intelligent transport system	Machine learning, IoT	Yes	Yes	No collision Improved road transportation Improved safety
[39]	Congestion and pollution control in transportation	Deep learning, IoT	Yes	Yes	Improved pollution control Congestion control by time method and route transfer
[40]	Sustainable and safety in transportation	IoT and Machine learning	Yes	No	Effectively managed road safety, minor collision
[41]	Collision and pollution in traffic management	IoT and Neural Network	Yes	Yes	Consumed less energy collision control method
[42]	Intelligent, sustainable transport	Machine learning, Cloud, and IoT	Yes	Yes	Smart route discovery zero collision
[43]	Green transportation	Neural Network, IoT	Yes	No	Pollution control method smooth traffic control
[44]	Pollution control and avoidance in transportation	IoT and Big data	Yes	Yes	Smart traffic lights and road pollution control
[45]	Smart transportation design	IoT, Machine learning	No	Yes	Smart city and parking system model
[14]	Safety issues in transportation	Big data, IoT	No	No	Road safety model analysis of accidental records identification of critical accidental zones
[46]	Smart parking	IoT, Machine learning	No	Yes	Smart city model
[47]	IoT Industry 4.0	IoT, Machine learning	Yes	Yes	Smart logistics and supply chain and automation in the industry
[48]	Pollution and smart transport	Cloud computing, IoT	Yes	No	Congestion control method and pollution control
[49]	Intelligent transport system	IoT and cloud computing	Yes	Yes	No collision improved road transportation
[50]	Automation in transportation	IoT and Machine learning	Yes	No	Improved pollution control congestion control improved time method and route transfer protocol

**Table 2:** Entities utilized in the proposed ATM system.

Entity	Subunit	Property	Functionalities
Vehicles	Automobiles (2, 3, and 4 wheelers)	Vehicle ID, speed, vehicle type, lane	To recognize a vehicle
	Vehicle control unit	Manual and automatic	To determine the vehicle control type
Infrastructure	Road unit	Lane ID, Lane name, length, one way, two way	To determine the road unit
	Traffic light control unit,	ID, installation status, delay duration	To determine the traffic light control unit
	Street light unit	ID, installation status	To determine the street light unit
Events	Vehicle to Vehicle Communication	Vehicle speed, vehicle turn information,	To determine the V2V communication
	Vehicle to infrastructure communication	Signboard, pedestrian crossing, traffic light, speed indicator	To determine the V2I communication

**Table 3:** Clustering Outcomes of DBSCAN and ML methods for accident detection.

Simulation Duration in Seconds	Vehicle Count(in Each Road Segment)	Cluster Type (Normal)	Cluster Type (Anomaly)
60	75	70	1
70	77	72	1
80	80	75	1
90	82	76	2
100	85	78	2
110	87	79	3
120	88	81	3
130	90	82	3

## Data Availability

The data that supports the findings of this paper is available from the corresponding author upon reasonable request.
